# Three-Dimensional *In Vitro* Cell Cultures as a Feasible and Promising Alternative to Two-Dimensional and Animal Models in Cancer Research

**DOI:** 10.7150/ijbs.96469

**Published:** 2024-09-30

**Authors:** Andrea Esposito, Alessandra Ferraresi, Letizia Vallino, Beatrice Garavaglia, Danny N. Dhanasekaran, Ciro Isidoro

**Affiliations:** 1 Laboratory of Molecular Pathology, Department of Health Sciences, Università del Piemonte Orientale, Via Solaroli 17, 28100, Novara, Italy.; 2 Stephenson Cancer Center, The University of Oklahoma Health Sciences Center, Oklahoma City, OK 73104, USA.

**Keywords:** tumor spheroids, organoids, microfluidics, organ-on-a-chip, 3D models, humanized mice

## Abstract

Cancer represents one of the diseases with the highest mortality rate worldwide. The burden of cancer continues to increase, not only affecting the health-related quality of life of patients but also causing an elevated global financial impact. The complexity and heterogeneity of cancer pose significant challenges in research and clinical practice, contributing to increase the failure rate of clinical trials for antitumoral drugs. This is partially due to the fact that preclinical models still present important limitations in faithfully recapitulating human tumors to serve as reliable indicators of drug effectiveness. Up to now, research and development strategies employ expensive animal models (including the so-called “humanized mice”) that not only raise ethical concerns, but also frequently fail to accurately predict responses to anticancer drugs because they do not faithfully replicate human physiology as well as the patient's tumor microenvironment. On the other side, traditional two-dimensional (2D) cell cultures fail to adequately reproduce the structural organization of tumor and the cellular heterogeneity found *in vivo.* The growing necessity to develop more accurate cancer models has increasingly emphasized the importance of three-dimensional (3D) *in vitro* cell cultures, such as cancer-derived spheroids and organoids, as promising alternatives to bridge the gap between 2D and animal models. In this review, we provide a brief overview focusing on 3D *in vitro* cell cultures as preclinical models capable of properly reproducing the tissue organization, biological composition, and complexity of *in vivo* tumors in a fine-tuned microenvironment. Despite their limitations, these models collectively enhance our understanding of the mechanisms underlying cancer and may offer the potential for a more reliable assessment of drug efficacy before clinical testing and, consequently, improve therapeutic outcomes for cancer patients.

## Introduction

Despite the development of improved prevention and early detection methods, as well as numerous advances in therapeutic strategies over the last decades, cancer remains one of the leading causes of death worldwide.^1^ Cancer is a multifactorial disease characterized by high complexity and heterogeneity, which makes experimental reproduction difficult and involves continuous therapeutic adaptations in the clinic.^2^,^3^ Due to cancer heterogeneity, a “one drug fits all” therapeutic approach is not feasible, as evidenced by the fact that current standard treatments are effective only in a subset of the patients' population. Tumors can exhibit intrinsic differences in the genetic background and may express different proteins in one patient compared to another. This variability highlights the urgent need for precision and personalized medicine.^4^ To this aim, the production of highly efficient and cost-effective drugs emerges as a crucial point in the future development of therapeutic approaches. Drug discovery is an arduous, meticulous, and expensive process; it is estimated that the cost of innovating and developing a new drug in the United States alone is more than $600 million, with an average timeline of 10-15 years.^5^ Therefore, there is a significant, unmet need to develop inexpensive and reliable preclinical platforms to accelerate the anticancer drug discovery pipeline to improve the outcome of cancer patients.^6^

Recapitulative disease models serve as important experimental tools, particularly in cancer research, where new drugs exhibit higher failure rates in clinical trials compared to other fields. In fact, only 5% of new anticancer compounds receive clinical approval, with the majority failing due to issues related to toxicity and insufficient effectiveness. This limited clinical efficacy can be partly attributed to the absence of preclinical models that closely reproduce tumor architecture, pathophysiology, and the crosstalk between cancer cells and the surrounding microenvironment. This represents the major shortcoming of the correlation between preclinical *in vitro* and *in vivo* results and the data obtained during the clinical trials.^7^,^8^ Therefore, a potential approach to mitigate the high attrition of anticancer drugs is to employ preclinical models that more closely represent *in vivo* human tumors, thereby obtaining more precise indications in terms of drug efficiency.^9^,^10^ To date, various models and technical approaches have been used to investigate and study the mechanisms underlying the key hallmarks of cancer.^2^,^11^

The two-dimensional (2D) *in vitro* cell cultures are traditional systems where cells grow as an adherent monolayer on a solid and flat plastic surface. These models have played a crucial role in setting the current knowledge of cancer biology research and continue to be widely used due to their high availability, easy manipulation, elevated level of reproducibility, and cost-effectiveness.^3^,^12^ However, 2D cancer cell cultures represent reductive models where cells are cultured under oversimplified and unrealistic conditions, thus failing to accurately mimic the real physiology of an *in vivo* tissue. They cannot reproduce the complex three-dimensional (3D) structure of a tumor or the dynamic interactions between cancer cells and the tumor microenvironment (TME). Moreover, cancer cells cultured as a monolayer on a flat plastic surface display significant alterations in crucial cellular signaling pathways and changes in their responses to stimuli. The 2D models then present further limitations: i) they do not preserve the real shape and polarization of cancer cells, ii) there is a marked lack of tumor cell heterogeneity, iii) they provide equal and unrestricted accessibility to essential nutrients, oxygen, growth factors, and metabolites, and iv) they offer an altered representation of critical cellular activities such as proliferation, migration, invasion, differentiation, gene and protein expression, response to stimuli and drug sensitivity. Furthermore, to maintain regular cell growth and ensure the supply of essential nutrients, adherent cells must undergo periodic trypsinization. This continuous procedure could lead to different and unpredictable genetic and epigenetic changes over time, consequently impacting cells' phenotype and behavior, such as growth and their response to both external and internal stimuli.^8^,^12^-^15^ Another aspect that limits the reproducibility of data in cultured cancer cultures is that the same cell line from different laboratories presents differences in its genetic and epigenetic background as a consequence of the long-term culture under different cultivation conditions, which ultimately leads to clonal evolution and selection of different subclones.^16^

Another widely employed tool in cancer research is represented by animal models, which act as an important link between *in vitro* studies and clinical experimentation.^17^ These models enable a deeper understanding of the complex nature of cancer biology and the implementation of novel strategies in terms of prevention, diagnosis, and treatment.^18^ Unlike 2D cell cultures, they effectively recapitulate the tissue structural organization and provide a system-level analysis.^19^ Specifically, mouse models represent the most commonly utilized *in vivo* systems due to their low cost, short gestation period, ease of genetic manipulation and the possibility to grow cancer cells from patients, generating patient-derived tumor xenografts (PDTXs). PDTXs can recreate the histological and molecular features, as well as the inter/intratumor heterogeneity of a patient's tumor, thus allowing the investigation of anticancer drug effectiveness. However, these models present several limitations ascribed to their dependence on immunodeficient hosts and the expensive costs related to their maintenance, long-term engraftment process, and molecular profiling. In addition, certain biological shortcomings hinder the efficacy and predictive value of murine models, as well as other types of *in vivo* studies. Genomic and pathophysiological differences between animals and humans allow the study of cancer hallmarks under conditions similar, but not identical, to those of patients. For instance, in the case of PDTXs, tumor engraftment might not occur, or metastatic patterns might not be faithfully replicated as in humans. Furthermore, the murine and human TMEs significantly differ, and mice also tend to substitute the exogenous tumor stroma and immune cells with their own. To overcome these limitations, humanized mice models consisting in severely immunodeficient mice (usually NOD-SCID) in which both patient's derived tumor cells and immune cells are co-engrafted could be employed. 20,21 One important drawback of these models is the lack of an adequate innate and MHC-dependent immune response and the inability to reconstruct a TME with a broad range of cytokines and stromal cells that can faithfully resemble that in the native patient's derived tumor. 21,22 This has relevance in terms of cancer stem cell renewal, angiogenesis, metastasization and tumor dormancy, all features that influence the natural growth and progression of the tumor and that impact the response to the therapy, thus affecting the translational value of immune-oncological research and drug testing in such animals. 21 Additionally, factors such as the gender, the number of animals enrolled in the study, their age, and the level of stress to which they are exposed can vary between laboratories and may significantly influence the outcome of the experiments.^8^,^19^,^23^ Considering these factors, the evaluation of drug effectiveness and toxicity in preclinical animal testing is not a foolproof indicator of its effects when translated to human clinical trials and raises ethical concerns as well.^24^ It is a fact that despite the preclinical test with these rodent models, more than 90% of drugs are revealed to be toxic to patients. 25

**Figure 1:**
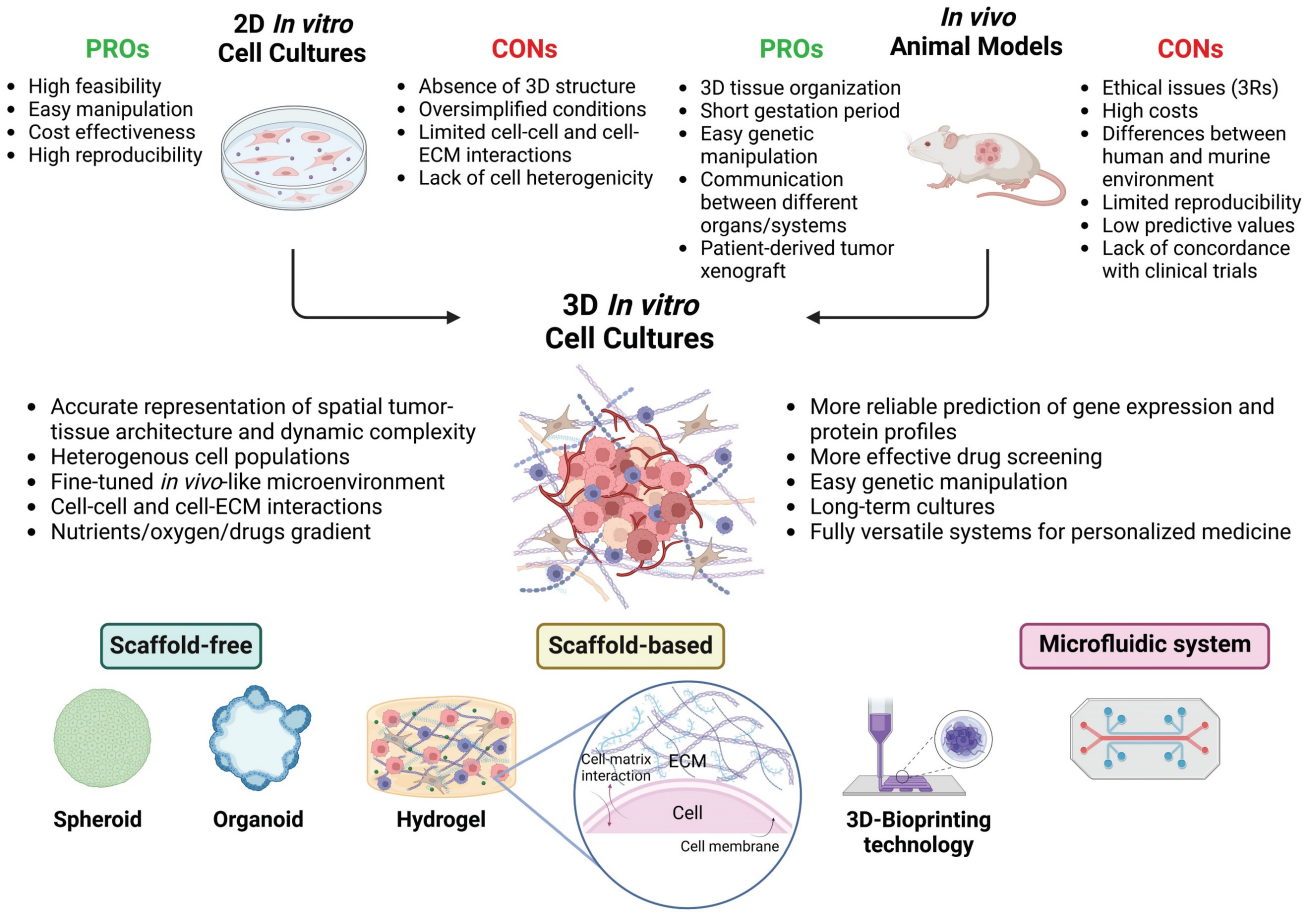
** 3D *In Vitro* Models: Bridging 2D Cell Cultures and Animal Models.** This image illustrates how 3D *in vitro* models bridge the gap between traditional 2D cell cultures and *in vivo* animal models. Unlike 2D cultures, which lack three-dimensional interactions and spatial organization, 3D cellular systems more accurately replicate the complex architecture and properties of tumor tissues. This realistic microenvironment enables precise studies of tumor biology and drug responses. In contrast to *in vivo* models, which may be limited by species-specific differences, 3D models can be tailored to closely mimic human tissues, enhancing their translational relevance and predictive accuracy. Ethically, 3D models offer advantages by reducing reliance on animal testing, adhering to the principles of the 3Rs (Replacement, Reduction, Refinement), and providing a more humane alternative for research. Various approaches can be used to develop 3D tumor models, including scaffold-free, scaffold-based, 3D-bioprinting, and microfluidic methods (created with Biorender).

The application of 3Rs' principle (e.g., Replacement, Reduction, Refinement) in research studies promotes a decrease or elimination in the use of laboratory animals, suggesting the need to develop and use new and more innovative models to overcome these limitations.^8^,^15^,^26^ In agreement, under recent legislation, the FDA (United States Food and Drug Administration) no longer requires animal testing to approve new drugs. Over the past decade, a pioneering approach to modeling cancer that incorporates the most recent advances in the fields of cancer research, pharmacology, tissue engineering, biomaterials, and nanotechnologies has progressively emerged. 3D *in vitro* cancer models are preclinical models that can accurately replicate the tumor-tissue architecture, biological composition, and dynamic complexity of *in vivo* tumors in a fine-tuned microenvironment. This facilitates a deeper comprehension of the mechanisms underlying tumor aetiology, growth, invasion, and metastasis, as well as the evaluation of drug efficacy before clinical testing.^27^ The growing need for more accurate cancer models highlights the relevance of these 3D cell culture techniques as a promising alternative for bridging the gap between 2D cell cultures and animal models (Figure 1). In fact, several 3D culture models have been developed and employed over the years.^28^,^29^

The focus of the present review is on the two widely employed 3D models in cancer research, namely spheroids and organoids, excluding the description of the methods by which they can be generated.

## Tumor Spheroids

Spheroids, first described by Sutherland and colleagues in the early 1970s, represent one of the most consolidated 3D culture methods for studying cancer biology. They are closely packed clusters of cells that faithfully reproduce several important properties of *in vivo* tumors, including structural organization, cellular heterogeneity, cell signaling pathways, deposition of the extracellular matrix (ECM), cell-cell and cell-ECM communications, growth kinetics, genomic and proteomic expression profiles, and pharmacological resistance.^30^,^31^

Spherical cancer models can be classified into different types based on cellular origin and methods of preparation, as widely described in the study published by Weiswald and colleagues.^32^ Among the reported models, multicellular tumor spheroids (MCTSs) emerge as the most extensively characterized and widely employed for recapitulating a variety of solid tumors, including breast, cervical, colon, lung, pancreatic, prostate, and adrenal cancers 33-39, among many others. MCTSs are self-assembling cellular aggregates consisting of primary cells or cell lines and mimic the physiological architecture of the tumor mass *in vivo* with its metabolic and proliferative gradients, acting as a clinically significant model for drug resistance studies. Moreover, the cell clonality, ease of maintenance, and simplicity of genetic manipulation make the 3D model the most suitable approach for high-throughput anticancer drug screening.^40^-^42^

MCTSs formation can be easily obtained in non-adherent conditions (like ultra-low attachment plates, continuous agitation and/or centrifugation) or in the presence of an exogenous scaffold.^43^ Depending on ECM composition and cadherin types and concentration (which vary for different cell types), spheroids assembly requires cell-cell adhesion and/or cell-ECM interactions that are orchestrated from homophilic cadherin-cadherin binding, integrins-ECM interactions, and cytoskeletal proteins remodeling. 44 In scaffold-based approaches, cells can proliferate dispersed in, or they can adhere to, ECM-mimicking acellular hydrogels.^45^,^46^ The scaffold can influence the mechanical and biochemical signals, facilitates cell-cell and cell-ECM interactions, and mimics the hypoxic and nutrient deprivation conditions of the native TME. 47 These models are particularly suitable for those tumors characterized by abundant ECM deposition and strictly dependent on it for their growth *in vivo*, like breast 48, pancreatic 49, lung 50 and liver cancers 51, for culturing primary patient-derived cancer cells, and for drug screening purposes. 52,53 In contrast, scaffold-free techniques mainly consist of culturing the cells under conditions that promote strong cell-to-cell interactions, facilitating cancer cell aggregation and ECM deposition. This method represents an excellent *in vitro* system for studies on tumor-specific processes like angiogenesis, invasion and metastasis. 54 Additionally, scaffold-free approach is a suitable platform to model the transcoelomic growth of peritoneal tumors (e.g., ovarian and colorectal cancers). 54-57

Tumor spheroids can be distinguished as homotypic, when composed exclusively of tumor cells, or heterotypic, when tumor cells are co-cultured with stromal cells, such as fibroblasts, endothelial cells, and immune cells.^31^,^58^ Remarkably, heterotypic spheroids allow to dissect the dynamic interactions between cancer cells and the cellular components of the TME, which play a crucial role in cancer metabolism and response to therapy. For instance, a heterotypic spheroid model (combined of pheochromocytoma cells and primary cancer-associated fibroblasts (CAFs)) was used to test the differential response of wild-type and SDHB/SDHD knock-down pheochromocytoma cells to the pro-migratory factors released by CAFs.^59^ To be noted, the 3D heterotypic spheroids displayed increased tumorigenic potential in terms of migratory capabilities compared to that of 3D homotypic cancer spheroids. 60 Giustarini and colleagues used 3D heterotypic tumor spheroids made of pancreatic ductal adenocarcinoma (PDAC) cells, endothelial cells, pancreatic stellate cells (PSC), and monocytes, which resembled some critical features of patients' PDAC immune microenvironment (e.g., immunosuppressive phenotype), for testing chemo- and immuno-therapeutics. 37

The employment of microfluidic systems or 3D-bioprinting technologies enable the development of even more sophisticated 3D cancer models, generating systems with complex architecture, physiological microenvironmental conditions, vasculature-like perfusion, precise control over chemical gradient flows, and mechanical forces.^15^,^40^,^61^ These techniques are also applicable for establishing organoids.

**Figure 2:**
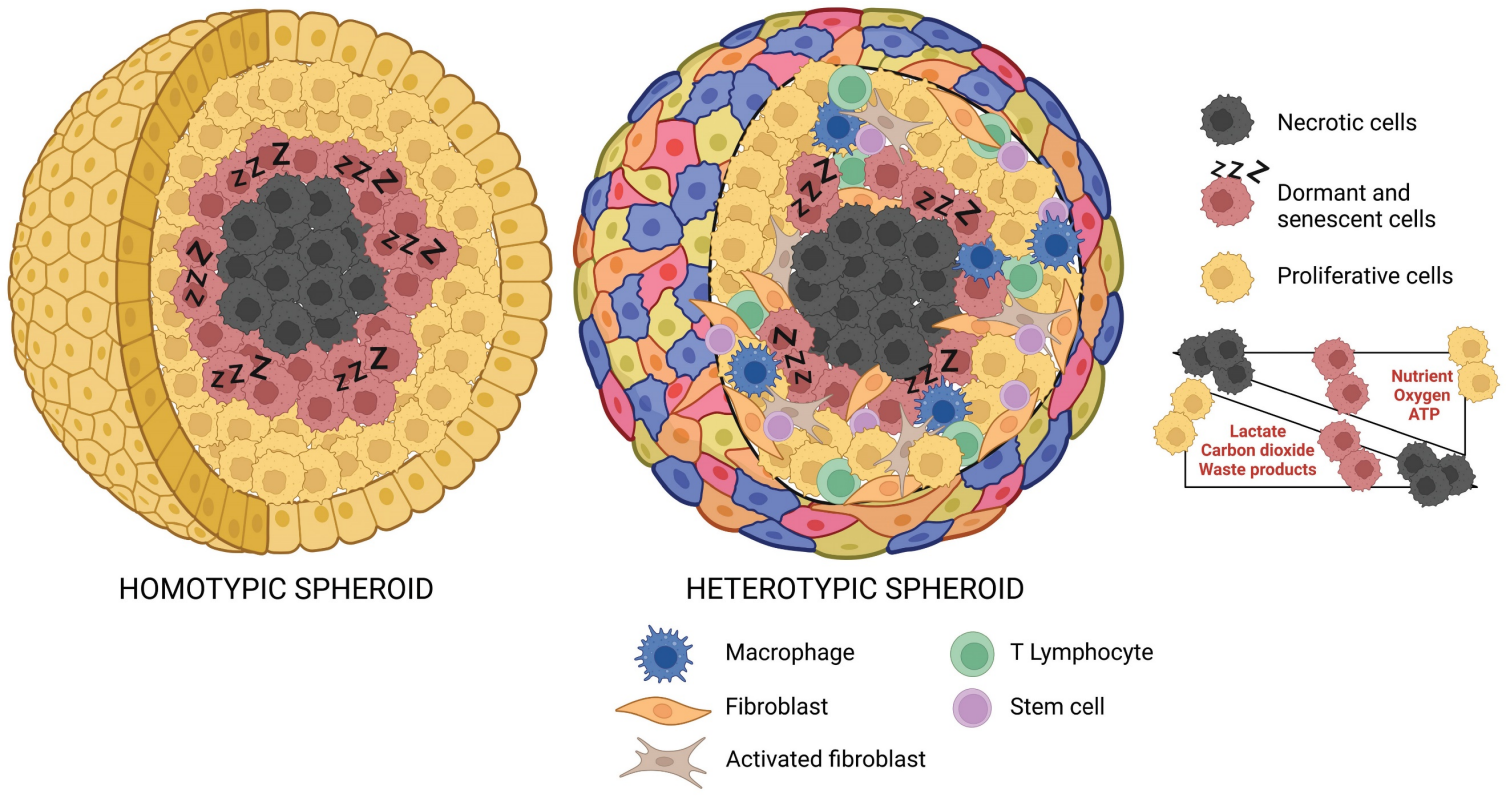
** Spatial Organization of Homotypic and Heterotypic Tumor Spheroids.** This schematic representation illustrates the 3D architecture of both homotypic and heterotypic tumor spheroids. The homotypic spheroid is comprised of a single cancer cell type, while the heterotypic spheroid includes multiple cell types, reflecting the complex nature of the tumor microenvironment. Both spheroids exhibit distinct regions: the outer proliferative zone, the intermediate quiescent (dormant) zone, and the central necrotic core. The figure also highlights the gradient distribution of essential factors such as nutrients, oxygen, lactate, carbon dioxide, and waste products across these zones, providing insights into the varying conditions within different regions of the spheroid (created with BioRender).

Tumor spheroids with a diameter exceeding 500 µm accurately reproduce avascular tumors or micrometastases, displaying a stratified structure with cells having various phenotypic, functional, and metabolic behaviors. Specifically, these spheroids present a well-organized spatial architecture that comprises an outer proliferative layer, an intermediate zone composed of quiescent and senescent cells, and an inner apoptotic and necrotic core resulting from the altered distribution of nutrients and oxygen (Figure 2). Typically, the diffusion gradient is confined to a range of 150-200 µm. 62 Indeed, the progression towards the spheroid core leads to a reduction of oxygen, nutrients, and pH levels, together with an increase of carbon dioxide, lactate, and waste products.^2^,^3^,^63^,^64^ Tumor cells can adapt to hypoxic conditions, by shifting from oxidative phosphorylation to anaerobic glycolysis and converting pyruvate into lactate to obtain energy. As a result, the release of lactate increases the acidification of the spheroid's inner regions.^65^,^66^

The structural organization and physiological characteristics of tumor spheroids strongly influence the response to therapy, the cell signaling pathways, and the profiles of gene and protein expression.^31^ Recently, our group demonstrated that the expression of the lysosomal protease cathepsin D (CD) is differentially modulated between 2D and 3D cell culturing, and this reflects on the survival efficiency of neuroblastoma (NB) cells. 67 Interestingly, CD-overexpressing NB cells were favored to grow in suspension (3D), while the CD knocked-down cells were favored for the growth in 2D, and when cells were switched from 2D to 3D and back to 2D culture conditions the surviving clones adjusted the expression of CD accordingly. 67 This example highlights how the culture condition influences the gene and protein expression.

Tumor spheroids can be exploited also to study *in vitro* the phenotypical changes and cancer cell behavior in relation to microbiota/host interactions. For instance, 3D colorectal cancer spheroids allowed to study how pro-inflammatory cytokines and probiotics impacted on cell proliferation and migration. 68,69

The hypoxic environment within tumor spheroids can induce chemo-radio resistance through various mechanisms. Firstly, hypoxic conditions cause an increase in hypoxia-inducible factors (HIFs) levels. Consistently, the expression of HIF-1α protein was detected in HeLa spheroids but not in the 2D culture counterpart.^70^ HIFs can induce the upregulation of the multidrug resistance (*MDR1*) gene, which encodes P-glycoprotein (P-gp), a cell membrane protein that actively pumps drugs or other molecules outside the cell. One study observed that the expression of HIF-1 in MCF-7 breast cancer spheroids caused an upregulation of P-gp, which, in turn, reduced the accumulation of doxorubicin within the spheroids; in contrast, no significant changes in HIF-1 and P-gp expression were observed in 2D-cultured cells.^71^ Furthermore, HIF-1 can regulate the expression of the vascular endothelial growth factor (VEGF), which is highly expressed within the hypoxic regions of spheroids where it contributes to drug resistance mechanisms.^72^,^73^ A375 melanoma spheroids were shown to exhibit higher expression of HIF-1 and VEGF compared to the same cells cultured as monolayers, and this variation affected the sensitivity of the cells to vemurafenib.^73^ It is known that hypoxia can impair the effectiveness of radiotherapy because DNA lesions, derived from reactive oxygen species (ROS) generated during water radiolysis, react with oxygen to generate stable DNA peroxides. This phenomenon contributes to pronounced radio resistance in tumor cells located in the inner part of the spheroid.^74^,^75^ Lastly, the hypoxic conditions within the spheroid are deleterious for drugs that cause cell membrane and DNA damage through the generation of ROS, such as doxorubicin 38,76,77 and cisplatin. 78 Moreover, drugs targeting highly proliferative cells (including carboplatin, cisplatin, doxorubicin, oxaliplatin, methotrexate, and paclitaxel) present limited efficacy against senescent and necrotic cells located in the inner regions of the spheroids.^79^ For instance, compared to 2D cell cultures, dense spheroids of BT-549, BT-474, and T-47D breast cancer cells showed increased resistance to doxorubicin and paclitaxel associated with elevated levels of hypoxia, high proportion of G0 dormant cells, and reduced expression of PARP and caspase-3. 33 In addition, low pH affects the efficacy of several anticancer compounds, such as doxorubicin, vinblastine, methotrexate, and anthraquinone, by impairing intracellular uptake.^80^-^82^

Finally, the strong E-cadherin-driven interactions between cancer cells and the secretion of ECM proteins (collagen, fibronectin, laminin, elastin, tenascin) determine an increase of spheroid density, forming a physical barrier that hampers the transport of therapeutic agents into the spheroid mass. Simultaneously, the elevated interstitial fluid pressure hinders the intake and distribution of antitumoral compounds.^10^,^83^,^84^ Several studies reviewed by Nunes et al. have shown that E-cadherin expression is more pronounced in spheroid cultures than in 2D cell cultures.^10^ Additionally, to confirm the role of E-cadherin on drug resistance in spheroids, an anti-E-cadherin monoclonal antibody (SHE78-7) was utilized to inhibit its function. The study shows that the administration of SHE78-7 increases the intracellular accumulation of chemotherapeutics (5-fluorouracil, etoposide, paclitaxel, and vinblastine) in HT-29 human colorectal cancer spheroids.^85^

The different gene and protein expression between 2D and 3D models provides an explanation for the distinct behaviors observed in 3D-cultured cells regarding growth, proliferation, migration, invasion, and drug sensitivity, when compared to cells grown in 2D.^46^ For instance, in a monolayer, several genes that promote growth and proliferation are frequently upregulated in comparison with their corresponding tissue origins, while genes that restrain these phenotypes tend to be suppressed. Therefore, cells grown in 2D cultures generally display a higher rate of proliferation in contrast to cells cultivated in 3D models. Although cells lose many of their original features when extracted from the primary tumor and cultured in a 2D system, the reintroduction of these cells into an *in vivo*-like environment largely restores the original features in terms of morphology, proliferation, and gene/protein expression.^86^,^87^ Differences in gene and protein expression profiles between 2D and 3D cultures have been reported for various types of cancers, including melanoma 88, colorectal cancer 89, mesothelioma 90, liver hepatocellular carcinoma 91 , and neuroblastoma 67. Ghosh and colleagues performed a comparative analysis of the expression patterns of 179 genes responsible for encoding chemokines, pro-angiogenic factors, and cell-adhesion molecules in both 2D and 3D models of melanoma cells. They detected significant upregulation of several genes that play important roles in promoting the progression, invasion, and metastasis of skin cancer in the 3D spheroids.^88^

miRNAs, which are key regulators of gene expression, can also be modulated in 3D models.^92^ Extracellular vesicles (EVs) released from 3D spheroids display differences in terms of secretion dynamics and molecular components (RNA and DNA) compared with EVs derived from 2D monolayers.^93^ In this context, miRNA expression profiles of EVs derived from 3D cultures of HeLa cervical cancer cells closely resembled those of circulating EVs isolated from the plasma of cervical cancer patients, with a high similarity of about 96%, in contrast to the miRNA expression patterns of EVs obtained from 2D cultured HeLa cells. Furthermore, culture and growth conditions had no impact on the genomic information carried by EVs, as demonstrated by DNA sequencing analysis.^93^ In addition, 3D models were useful for investigating miRNA-mediated regulatory mechanisms in ovarian cancer. Yoshimura and colleagues studied the effect of miR-99a-5p, a microRNA overexpressed in epithelial ovarian carcinoma (EOC), on peritoneal dissemination. They utilized human peritoneal mesothelial cells (HPMCs) treated with EOC-derived exosomes. Results revealed that upregulation of miR-99a-5p in HPMCs promoted EOC invasion by inducing upregulation of fibronectin and vitronectin, suggesting its potential utility as an EOC biomarker in serum and as a potential therapeutic target.^94^ 3D ovarian cancer spheroids, mimicking peritoneal metastases dissemination, have been employed in our laboratories to study autophagy-dependent cancer cell dormancy and how this was interrupted by IL-6-mediated upregulation of the oncomiRNA miR-1305.^95^ Furthermore, the expression of miRNAs in 3D cultures compared to 2D cultures has been investigated in different breast cancer cell lines. Nguyen and colleagues examined the miRNA expression profile in 3D cultures *versus* 2D cultures in the breast cancer cell lines MCF-7 (non-invasive) and MDA-MB-231 (invasive). They found that 49 miRNAs exhibited differential expression in the MCF-7 cell line when cultured in 3D compared to 2D, while in the MDA-MB-231 cell line 28 miRNAs displayed differential levels.^96^

Finally, differences in crucial cellular signaling pathways may exist between 2D- and 3D-cultured cells. Compared to their 2D counterparts, colon cancer cell lines cultured in a 3D environment exhibit a reduced activation of the AKT/mTOR/S6K pathway, which plays an important role in carcinogenesis, cancer cell migration, and resistance to therapies.^97^ Similarly, 3D cultures of ER^+^/Her2^+^ breast cancer cells were less responsive to hormonal and anti-HER2 treatments compared to 2D cultures due to a shift from the AKT to the MAPK pathway.^98^

Recently we have proposed a variant of heterotypic/homotypic spheroids made of a mixture of subclones of cancer cells genetically engineered to express different levels of a protein as an *in vitro* model that could *bona fide* represent the genetic changes occurring during clonal evolution. 67 The model allowed to determine which clone, among the ones hyper-expressing or silenced for cathepsin D, would overtake the other depending on the culture condition.

## Tumor Organoids

Organoids are miniaturized replicas of tissues or organs, originating from stem cells with the ability to differentiate and self-assemble into *in vitro* 3D structures, faithfully mimicking the morphology and functionality of their *in vivo* counterparts.^99^-^100^ Organoids can be derived from various types of stem cells, including induced pluripotent stem cells (iPSCs), adult stem cells (ASCs), or embryonic stem cells (ESCs).^14^,^101^ Like spheroids, organoids are cultured in the presence of an ECM-mimicking scaffold that provides mechanical support to the cells.^29^ Clevers and his group pioneered the development of organoids by using single mouse intestinal ASCs under specific culture conditions that could reproduce the *in vivo* stem cell niche, thereby inducing the proliferation and differentiation of the intestinal crypt epithelium.^102^

Organoid cultures have been established for several healthy and cancer tissues, such as colon, breast, liver, lung, pancreas, prostate, ovary, among others. 3,103,104 The employment of patient-derived tumor samples has led to the creation of the well-known model commonly referred to as “tumoroid”. 3,103,104

Tumoroids include different cellular subpopulations with specific genetic alterations, thereby maintaining the cancer heterogeneity normally found *in vivo*. They also retain the genetic signature of the host patient, making them a predictive platform for studying patient response to treatment, guiding the decision-making processes, and improving the effectiveness and efficiency of clinical studies.^101^ Tumoroids can be developed by engineering healthy organoids using genetic editing technologies such as CRISPR-Cas9, allowing a deep investigation into the onset and progression of tumors as well as testing new therapeutic tools.^105^ An engineered gastric cancer organoid model with and without AT-rich interactive domain 1A (*ARID1A*) mutations has been employed to address the context-dependent role for *ARID1A* in early neoplastic transformation and to identify genotype-dependent therapeutic vulnerabilities. 106 Even more complex models of human colon organoids, recreating the sequence with up to five different oncogenic mutations in *APC*, *KRAS*, *TP53*, *SMAD4* and PI3K catalytic subunit-α (*PIK3CA*), has been utilized for testing drug response. 107,108

Disease modelling in animals is limited by interspecies differences, a limit that can be overcome by human-derived organoids. Additional advantages of organoid cultures include their ability to be expanded *in vitro* and maintained genetically and phenotypically stable over prolonged periods. Moreover, they can undergo genetic modifications and cryopreservation, facilitating the establishment of an organoid biobank for preclinical studies, encompassing various cancer types directly derived from patients.^103^,^104^,^109^,^110^ Furthermore, the generation and maintenance of organoids represent significantly more efficient and economically advantageous processes, compared to PDTX models.^111^

Overall, these characteristics consolidate organoids as an optimal model for personalized cancer medicine.

The ability to model healthy and cancerous tissues simultaneously from the same patient stands out as an important advantage of organoids, providing an effective tool for drug screening. This model aids in the identification of compounds that selectively target cancer cells over healthy ones, allowing the selection of less toxic substances and, consequently, reducing the risk of side effects.^103^,^112^ Additionally, the development of organoids from different tumor regions of the same patient enables the reproduction of the tumor heterogeneity paving the way for a personalized therapy.^42^ In 2015, van de Wetering and colleagues established the first organoid biobank originating from colorectal cancer patients. The biobank included 20 primary tumors together with organoid cultures derived from adjacent normal tissues. By performing a high-throughput automated drug screening, they evaluated several different compounds, including conventional chemotherapeutics and novel targeted inhibitors, on the complete organoid panel. Subsequently, the drug sensitivity data were correlated with the genomic features of cancers to identify molecular signatures and clinically relevant biomarkers associated with treatment responses.^113^ In another study, Pauli and collaborators generated tumoroids and corresponding PDTX models from patients affected by different malignancies. Their comparative study revealed histopathological similarities between the latter models and their parental tumors, further validated through whole-exome sequencing.^114^ The analysis demonstrated that tumoroids preserved the genomic alterations of the tissue of origin during prolonged culture. Furthermore, genome sequencing of numerous tumor samples showed that 85.8% of cases had non-targetable somatic alterations in cancer genes, 9.6% could be affected by off-label drugs, and only 0.4% of identified somatic alterations were susceptible to FDA-approved drugs.^114^ These findings highlight the potential of reliable cancer models in unveiling new therapeutic options. Screening of 160 drugs, including FDA-approved chemotherapeutics and targeted compounds, showed comparable drug responses between tumoroids and PDTX models.^114^ Moreover, to comprehensively encompass the diversity of breast cancer, a biobank comprising over 100 organoids derived from both primary and metastatic breast cancers has been established.^115^ These tumoroids faithfully mimic the typical morphology and histopathology of breast cancer while largely conserving the hormone receptor and Her2 status of the parental tumors. This facilitated *in vitro* drug screening that corresponded to patient responses.^115^ Finally, among the various successfully developed biobanks, two organoid platforms have been established, derived from multiple stages and subtypes of ovarian cancer, accurately reflecting intra- and inter-patient heterogeneity. The pharmacological analysis conducted on these organoids included both chemotherapeutics (platinum/taxanes) and targeted agents (PI3K/AKT/mTOR inhibitors or PARP inhibitors), revealing significant differences in drug sensitivity that strongly correlated with clinical responses.^116^,^117^

Organoids faithfully replicate the genomic and transcriptomic profiles of the patient, aiding in the identification of potential prognostic biomarkers. In a study, bladder cancer organoids exhibited high concordance with the mutational profiles of the parental tumors, including mutations in genes such as *TP53, RB1, FGFR3*, or epigenetic regulators, like *ARID1A, KMT2C, KMT2D*, and *KDM6A*.^118^ In another study, early cultures of liver tumoroids (less than 2 months) maintained approximately 92% of each patient's genetic variants, with over 80% of the genetic variants retained even in more advanced organoid cultures (beyond 4 months).^119^ Additionally, a comparison of the transcriptomes of all primary liver cancer organoids with those derived from healthy livers resulted in the identification of novel prognostic markers. Specifically, this analysis revealed that the overexpression of *C19ORF48*, *DTYMK*, or *UBE2S* in hepatocellular carcinoma and the overexpression of *C1QBP* in cholangiocytes were associated with an unfavorable prognosis.^119^ Another study highlighted a high concordance, even after long-term culture, in terms of genomic and transcriptomic profiles between gastric tumoroids, encompassing different subtypes, and the corresponding *in vivo* tumor tissues.^120^ In addition to the genomic signature, tumoroids faithfully conserve the specific epigenomic features of the modeled tumor type.^111^ The analysis of the DNA methylation profile in colon organoids cultured *in vitro* for a prolonged period (12-14 months) revealed the occurrence of spontaneous promoter hyper-methylation, reminiscent of an aging-like process.^121^ This epigenetic modification resulted in the silencing of pivotal genes in the Wnt signaling pathway, subsequently leading to a progenitor-like cellular state susceptible to neoplastic transformation through the Braf^V600E^ mutation. In contrast, the absence of such promoter hyper-methylation in short-term cultured organoids resulted in significantly greater resistance to Braf^V600E^-induced transformation.^121^ Indeed, the transformation of long-term cultured organoids took only two weeks, while five months were required for the younger organoids. It is noteworthy that the CRISPR/Cas9-mediated editing of important genes in the Wnt signaling pathway, targeted by DNA hypermethylation, successfully reproduces the aging-like spontaneous epigenetic silencing.^121^

Organoids also stand out as a valuable *in vitro* platform for exploring the expression profile and the functional role of miRNAs. In a study that examined the role of miRNAs associated with the early stages of tumorigenesis in murine intestinal tumor organoids, microarray analyses revealed a pronounced downregulation of specific miRNAs, such as miR-194 and miR-215, in intestinal tumor organoids compared to those derived from normal intestinal epithelium. Specifically, the enforced expression of miR-194 resulted in the inhibition of a key positive regulator of the cell cycle, E2F3, thereby suppressing the growth of intestinal tumor organoids.^122^ Furthermore, the forced expression of miR-215 was able to suppress the cancer stem cell signature by decreasing the levels of intestinal stem cell markers, including LGR5.^122^

Another possible application of organoids is the study of host/pathogen interactions. Clevers and his group has implemented a 3D model to assess the oncogenic potential of *Helicobacter pylori* in human and murine gastric organoids. This platform can be adapted to other tumors and different bacteria, viruses or parasites, thus allowing *in vitro* investigation of infection-induced changes in primary cells. 123 Rao and co-workers reported a 3D organoid platform to assess the effects of HBV infection on liver tumorigenesis. Liver organoids retain *in vivo* features and are able to support the complete HBV replication cycle, thus paving the way for design novel HBV-target therapies. 124 Another study by Toyohara and colleagues established squamocolumnar junction organoids to study the molecular mechanisms involved in HPV18-related cervical carcinogenesis. This model allows to identify novel genes involved in HPV18 early promoter activities, which might serve as therapeutic targets in HPV18-infected cervical lesions. 125

Although organoids have gained considerable importance in both basic and translational cancer research, there are still technical and scientific challenges that need to be addressed to fully exploit their potential. Firstly, pronounced variability exists in the success rates of organoid culture, both across different cancer subtypes and within distinct samples of the same tumor type. Organoids modeling, particularly for specific tumor types, can pose intricate challenges. Secondly, different studies on cancer organoids introduce substantial technical variability due to the use of non-standardized and inadequately defined culture protocols, including variation in the source of tumor tissue, culture medium formulations, and the use of animal-derived 3D matrices. This variability, in turn, translates into an inaccurate representation of the biological heterogeneity of cancer, potentially affecting drug development and the identification of biomarkers. Thirdly, the initial growth of non-malignant epithelial cells, identified and confirmed solely through genomic sequencing, must be considered. Lastly, tumoroids fail to capture the TME, as they often exclusively include cancer cells and lack other essential cell types such as fibroblasts, immune cells, and endothelial cells. These cells regulate biological processes like cell proliferation, ECM production, vascularity, angiogenesis, and anti-tumor immunity, driving drug response and tumor aggressiveness. However, this limitation could be overcome through the establishment of a co-culture system.^3^,^40^,^101^,^111^,^126^ Colorectal cancer organoids, when cultured in isolation, display a deficiency in gene expression associated with cell-to-cell communication with the TME, a crucial feature present in the cancer tissues of origin. However, upon co-culturing colorectal cancer organoids with CAFs, a patient-dependent re-expression is observed in various genes originally present in the tumor tissue and recognized for their oncogenic functions. This serves as compelling evidence that the microenvironment actively contributes to the regulation of processes involved in tumor progression, such as differential gene expression.^127^

Another important point to consider is the difficulties to completely reproduce within the tumoroid the complex dynamic of the immune environment. This is relevant in view of the importance that immunotherapy has nowadays. 128 However, attempts in this sense are being made by co-culturing organoids with peripheral blood mononuclear cells (PBMCs) or immune cells from lymph nodes and CAFs to create a TME in which tumor cells are embedded in cytokines and all types of immune cells. 129

Moving forward, there are several opportunities to develop more sophisticated models that can better mimic *in vivo* conditions. Tumor organoids have been integrated with advanced technologies such as 3D-bioprinting and/or microfluidic chip systems. The combination of microfluidic devices with organoids results in “organoids-on-a-chip”, offering a unique approach to investigate tumor-stroma interactions and their systemic effects. On other hand, combining 3D-bioprinting with organoids ensures the proper spatial arrangement of cells within intricate 3D constructs and preserves the hierarchical architecture of the TME, and enhances model reproducibility.^15^

## Exploring the Potential of Microfluidics in Clinical Research and Medicine

Undoubtedly the 3D *in vitro* culture systems helped to bridge the gap between traditional 2D cell cultures and animal models by exploiting the strengths of the two approaches. While 3D models strike a balance between ease of manipulation and closer representation of *in vivo* physiology, they still fall short of completely replicating the *in vivo* scenario. This limitation is attributed to the absence of native physiological stimuli that cells experience in their living microenvironment.

Within 3D tissues, a network of arteries, veins, and capillaries ensures continuous perfusion of oxygen and nutrients, as well as of drugs, throughout entire organs. The spatial organization plays a crucial role, exposing cell populations to varying gradients of substances that significantly influence the phenotypic and metabolic behavior of the tissues.^46^,^130^ Fluid flow is thus considered the part and the parcel of the tissue architecture. The fluidic system, influenced by vessel type, tissue structure, and organism size, generates physiological shear stress impacting the morphology, viability, proliferation, differentiation, and gene expression of a cell and its interactions with other cell populations. These effects are observed not only under normal conditions but also in pathological states.^131^-^133^ Immune cells exploit the systemic circulation to identify the regions of the organism affected by inflammation or damage, with their extravasation typically occurring in the vessel walls experiencing higher shear stress.^134^ Additionally, shear stress plays a pivotal role in regulating cancer invasion and metastasis spread. On one hand, cancer cells utilize systemic circulation to disseminate and colonize distant organs, especially during intra- and extravasation processes. On the other hand, the shear forces within the vessels may hamper the survival of invading cancer cells, hindering their successful metastasis.^131^,^135^

**Figure 4:**
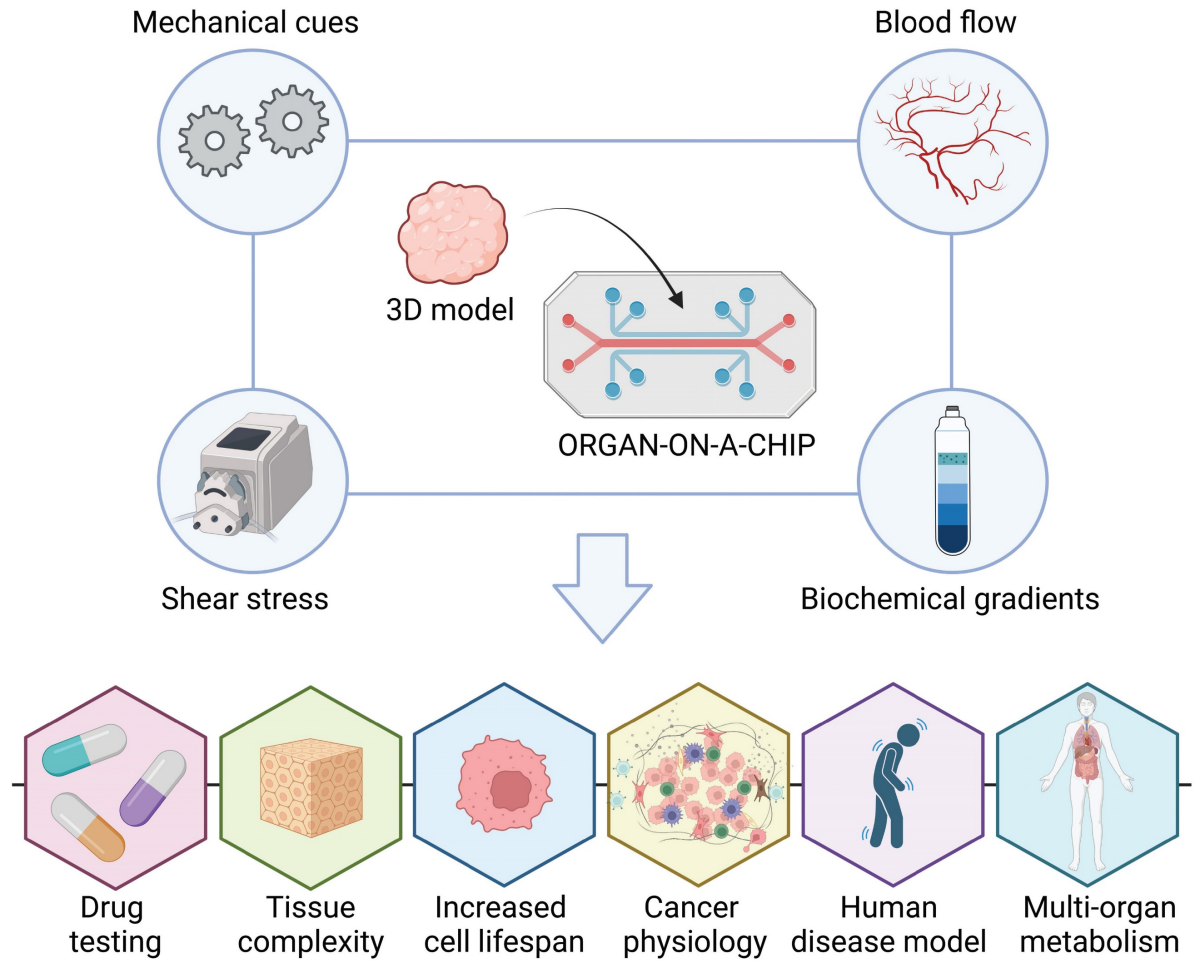
** Microfluidic Devices: Advancing 3D Tissue Models Towards Organ-on-a-Chip Technology.** Microfluidic devices have recently gained widespread application in clinical research to address the limitations of static 3D conditions while adhering to the principles of the 3Rs. 3D models with microfluidics have led to the development of an “organ-on-a-chip concept”. These systems provide a practical platform for simulating *in vivo* body fluid perfusion, mimicking organ blood flow through externally controlled microfluidics. This capability facilitates the recreation of biochemical gradients and mechanical cues essential for tissue function. “Organ-on-a-chip” enhances reproducibility of tissue conditions, extends cell lifespan, accelerates drug testing without animal models, and establishes reliable human disease models for basic research (created with BioRender).

This evidence highlights the importance of the fluid dynamics in the biological systems. Therefore, to address the above-mentioned limitations of 3D static conditions, while adhering to the 3Rs' principles, microfluidic devices have recently gained widespread application in clinical research.^136^ This promising solution involves integrating 3D models with microfluidics, giving rise to the concept of an "organ-on-a-chip” (Figure 4). This system provides a practical platform for simulating *in vivo* body fluid perfusion, enabling to mimic the blood flow in organs through externally controlled microfluidics, facilitating the recreation of biochemical gradients and mechanical cues.^137^ They enable the reconstruction of 3D living tissues under micro physiological fluid-dynamic conditions, facilitated by a peristaltic pump that replicates the speed, direction, and shear stress of fluid flow. This technology recapitulates diverse flows and tissue complexity 132,138, enhancing the reproducibility of tissue conditions, extending cell lifespan, accelerating drug testing without involving animal models, and achieving reliable human disease models for basic research.

Organ-on-a-chip technologies enable the comprehensive study of various aspects of cancer physiology. This includes the recreation of the TME, investigation of cancer-stroma and -immune crosstalk 139, analysis of cell migration and metastasis, screening of anticancer drugs, prediction of therapy responses, and exploration of the transport of anticancer nanomedicines in tumor tissues 140. As an example, creating 3D SKOV-3 cell-laden alginate hydrogels as models for ovarian tumors under fluid dynamic conditions provides a more precise representation of the 3D TME and improves the prediction of *in vivo* drug efficacy compared to static *in vitro* models and xenograft mouse models.^130^ After a week of treating 3D hydrogels with 10 μM cisplatin, cell viability exceeded 80% under static conditions but declined by up to 50% in dynamic culture, which reflected the different cisplatin diffusion rate in the two conditions. Notably, in a xenograft model, the drug efficacy test demonstrated around 44% tumor regression after 5 weeks, aligning with predictions from shorter-term fluid-dynamic *in vitro* tests.^130^

The immune-organ-on-a-chip replicates the infiltration of circulating immune cells into a 3D tumor model, offering dual access to both tumor and circulating compartments. This enables the monitoring and quantification of changes in the TME, including soluble molecules, cell death, and tumor cell invasion. Within the microfluidic device, circulating NK cells undergo a spontaneous extravasation process, retaining their ability to interact with matrix-embedded neuroblastoma cells, thereby exhibiting a cytotoxic effect that leads to tumor cell apoptosis.^141^

Microfluidic devices are also employed in studying the metastatic dissemination of tumor cells, particularly for isolating circulating tumor cells (CTCs). The detection of CTCs serves as a predictive measure for tumor staging. CTC isolation utilizes label-free filtering methods 142,143 or label-based approaches involving specific antibodies.^144^ Researchers have replicated a 3D circulation system with different patterns of wall shear stress to investigate its effects on the behavior of circulating metastatic breast cancer cells injected into the device. 131

Organ-on-a-chip devices could also facilitate the intricate dynamics of multi-organ metabolism when interconnected in various organs-on-a-chip, as well as the pharmacokinetics 145, including the mechanism of action and toxic effects of drugs 146. A multi-organ chip was used to interconnect ovarian cancer tissues with hepatic cellular models, emulating systemic cisplatin administration. This setup allowed the simultaneous evaluation of drug efficacy and hepatotoxic effects in a physiological context. Notably, the combination of 3D culture, fluid-dynamic conditions, and multi-organ connection showcased superior predictive toxicity and efficacy results compared to clinical therapy. 147

In addition, microfluidic-coupled devices represent a valuable platform to be exploited in precision medicine, particularly for the preclinical screening of anticancer nano-drugs and nanotheranostic systems. 148

## Advancing Cancer Research through 3D-Bioprinting for Personalized Medicine

Despite significant progress in 3D co-culture and microfluidics, fully capturing the complexity of *in vivo* tumors remains a major challenge. The TME consists of a complex array of elements, including the ECM, stromal cells, immune cells, soluble mediators, and blood vessels. 3D-bioprinting enables the precise replication of this intricate architecture by creating tissues and organs that faithfully reproduce the cellular density, ECM composition, and three-dimensional spatial organization of the TME. 15 In recent years, 3D-bioprinting has emerged as an innovative approach for fabricating complex tissue models by producing biomaterials through designed structures. These biomaterials possess powerful properties such as biocompatibility, controllability, printability, and crosslinking, enabling diverse applications in regenerative medicine, cancer research, drug discovery, toxicology, and basic research. 149 For the successful implementation of 3D-bioprinting technology, the use of bioinks is fundamental, as they have recently emerged as indispensable for achieving fast and dependable 3D-bioprinted cell culture systems. 150 Bioinks consist of biocompatible hydrogels embedded with various forms of living cells, including single cells, cell aggregates in spheroids, and cells organized into organoids. 150,151 These biopolymeric hydrogels should replicate the structural, physicochemical, and biological characteristics of the ECM, enhancing the simultaneous attachment and proliferation of various cell types. 152 Additionally, bioinks may include biomolecules such as growth factors, DNA, miRNA, cytokines, and exosomes. 150 By using the bioprinter, hydrogels, cells, and biomolecules can be precisely layered and arranged to control the spatial placement of functional components, enabling the creation of customized 3D structures. 153

The combination of organoids with 3D-bioprinting offers a promising avenue for developing more advanced and accurate cancer models, focusing on clinical applications such as chemotherapeutic drug screening and the development of personalized treatment regimens for cancer patients. 15,61

Bioprinted cancer models integrating patient-derived cancer cells and stroma components to mimic the TME and vascularization offer advanced platforms for precision chemotherapy screening across various cancers. For example, Han et al. developed a bioprinting method to recreate the TME by printing a layer of blood vessels using fibroblasts and endothelial cells within a biocompatible matrix. The glioblastoma tumor spheroids were added, and the formation of blood vessel sprouts around the spheroids was observed, which increased their size. Treatment with the drug temozolomide effectively reduced spheroid growth, demonstrating that this bioprinted model is a valuable tool for studying tumor biology and testing drug efficacy. 154 A high throughput bioprinting platform using tunable 3D hydrogels proved effective for testing anti-metastatic drugs toward cancer cells exhibiting distinct migratory and invasive behaviors in dependence of the hydrogel stiffness. 155

Altogether, these models hold promise for personalized medicine by closely mimicking *in vivo* conditions and improving treatment strategies. 156

Furthermore, the integration of 3D-bioprinting with innovative technologies, such as organ-on-a-chip systems, is likely to revolutionize cancer research. Organ-on-a-chip devices, which mimic the physiological functions of human organs as mentioned above, can be combined with bioprinted tissues to create highly dynamic and interactive cancer models. 157,158

The continuous evolution of 3D-bioprinting technology offers a powerful means to replicate the intricate features of the TME, bringing us closer to creating more accurate cancer models. This progress aligns with the growing demand for prognostic preclinical models that identify the most suitable treatment regimens for individual patients, ultimately enhancing treatment efficacy and minimizing adverse effects, and thus advancing the field of precision medicine.

## Concluding Remarks

This review provides a comprehensive overview of primary models employed in cancer research, including *in vitro* cell cultures and animal models. Particular emphasis is placed on their distinctive characteristics, strengths, and limitations. Here, we meticulously examined the 3D *in vitro* cell cultures of spheroids and organoids in all their variant implementations, including microfluidic and bioprinting. We stress how these 3D models may represent valid alternatives to the oversimplified and unrealistic 2D *in vitro* models as well as to animal models, that cannot accurately replicate the complex human conditions. We have thoroughly discussed the advantages offered by tumor spheroids and organoids in comparison to 2D and animal models, particularly in drug screening, differential gene and protein expression, epigenetic regulation, cellular signaling, and biomarkers identification.

In our discussion, we highlight the strengths of 3D models, while acknowledging their limitations as summarized in Table 1.

**Figure 5:**
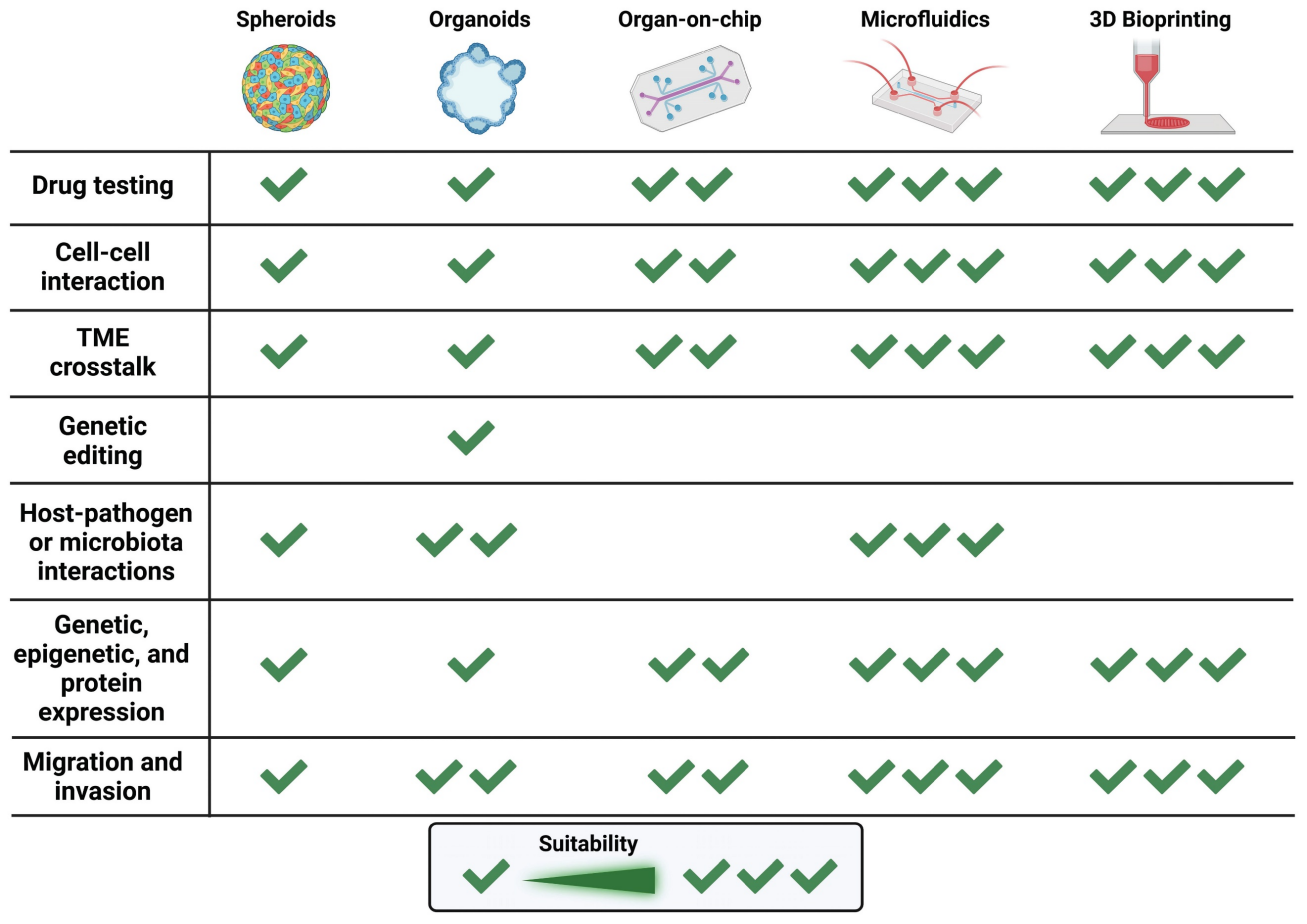
** Overview of the Current Applications of 3D Models in Cancer Research.** Schematic representation of the suitability of each 3D culture system (spheroids, organoids, organ-on-chip, microfluidics and 3D-Bioprinting) to study the onset and progression of carcinogenesis, efficacy of anticancer therapies, cell-cell crosstalk in the TME, host/pathogen or host/microbiota interplays, expression profiling, and metastatic process (created with Biorender).

The implementation with microfluidics holds promising potential in 3D models exploited in clinical research. Further investigations are essential to uncover new possibilities in microfluidic applications, potentially leading to more personalized and precise medicine. This evolving field has the capacity to drive breakthroughs in diagnostics, drug delivery, and tissue engineering, offering innovative solutions to complex challenges in diverse medical fields. A brief overview of the possible applications and advantages of all the 3D models mentioned above are illustrated in Figure 5.

Taken together, 3D models emerge as a pivotal tool bridging the gap between 2D *in vitro* and *in vivo* models. The increasing complexity of 3D cell cultures, resulted from the integration of spheroids and organoids with microfluidic systems and/or 3D-bioprinting, brings researchers closer to mimicking *in vivo* conditions. The employment of 3D models represents a significant advancement in *in vitro* research, contributing to the development of effective antitumor strategies. Their application promises a reduction in animal experimentation, offering advantages in terms of costs, time efficiency, and ethical considerations. Consequently, this advancement enhances the robustness and reliability of research data, facilitating the seamless translation of findings from the experimental phase to clinical applications. We are aware that *in vitro* models, although sophisticated and useful for understanding biological mechanisms, also present important limitations as they cannot reveal organ toxicity or provide information on pharmacokinetics, which can be derived from animal studies.^21^ Here we presented the bioengineered 3D *in vitro* models that could serve as valid alternative platforms to limit the use of animal models for preclinical testing.

## Figures and Tables

**Figure 3:**
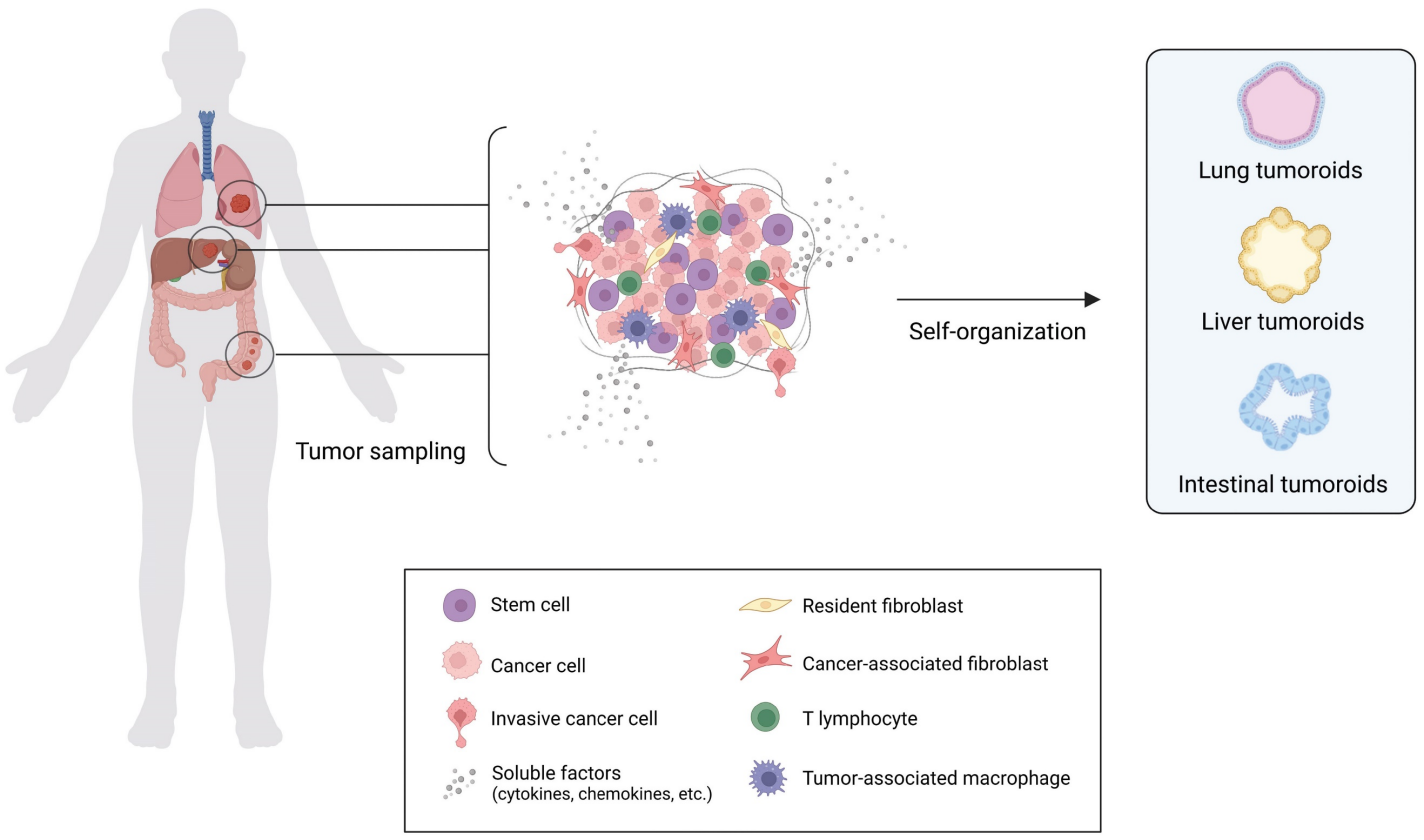
** Organization of Tumor Organoids.** Schematic representation of *in vitro* heterotypic organoids (refer as tumoroids) formation. Tumoroids are simplified versions of the patient-derived tumor mass containing stem cells that drive the self-organization in the 3D architectures capable of recapitulating several aspects of the complexity and functionality of the corresponding *in vivo* tissue (created with BioRender).

**Table 1:** Overview of the main advantages and limitations of 3D models discussed in the review. Abbreviations: ECM, extracellular matrix; TME, tumor microenvironment.

Experimental model	PROS	CONS
Spheroids	Low expensive and time effective (scalable in high throughput) Easy manipulation and high accessibility of cell materials High reproducibility and long-term growth Simplicity of genetic manipulation Possibility of setting up co-cultures for mimicking tumor heterogenicity	Oversimplified static system Lack of robustness and reliability for translational relevance Lack of ability to recapitulate *in vivo* organ features
Organoids	Faithfully recapitulate the pathophysiology of the disease Highly predictive platform for studying patient responses Accurately replicate tissue architecture, functionality and dynamic complexity of TME Simplicity of genetic manipulation Possibility of setting up co-cultures for mimicking tumor heterogenicity Possibility to model healthy and tumor tissues simultaneously from the same patient Development of biobanks	Challenging manipulation Need of ethical approval for patients-derived samples High expensive Limited growth (depending on stemness/differentiation ratio) Limited reproducibility Oversimplified static system
Organ-on-chip	Combination of advantages of microfluidic devices with organoids Possibility of interconnecting different organoids to recreate multi-organ metabolism (multi-organ-on-chip)	High expensive Require expertise Need of ethical approval for patients-derived samples Difficult to adapt to high throughput Readout typically limited to end-point analysis
Microfluidics	Replicate physiological TME conditions, vasculature-like perfusion, precise control over chemical gradient flows, and mechanical forces Possibility of collecting the fluids for characterization of secreted soluble factors and circulating cells Implementation of a dynamic system	Low amount of samples available for downstream characterizations Require expertise High expensive Difficult to adapt to high throughput
3D-Bioprinting	Faithfully recapitulate complex architecture, precise control over chemical gradient flows, and mechanical forces (stiffness) of native ECM Proper spatial arrangement of cells within intricate 3D constructs and preserves the hierarchical architecture of the TME High reproducibility	Require disruption of hydrogel/matrix to collect cells Low amount of samples available for downstream characterizations Require expertise High expensive Difficult to adapt to high throughput
